# Supplementary material for: Pleiotropic Effect of the compactum Gene and Its Combined Effects with Other Loci for Spike and Grain-Related Traits in Wheat
Source: Plants (Basel). 2022 Jul 13;11(14):1837. doi: 10.3390/plants11141837 (PMC9316965; doi:10.3390/plants11141837)
Supplement: Supplementary file 1 [file plants-11-01837-s001.zip › Supplementary Figures S1-S4.pptx]

## Slide 1
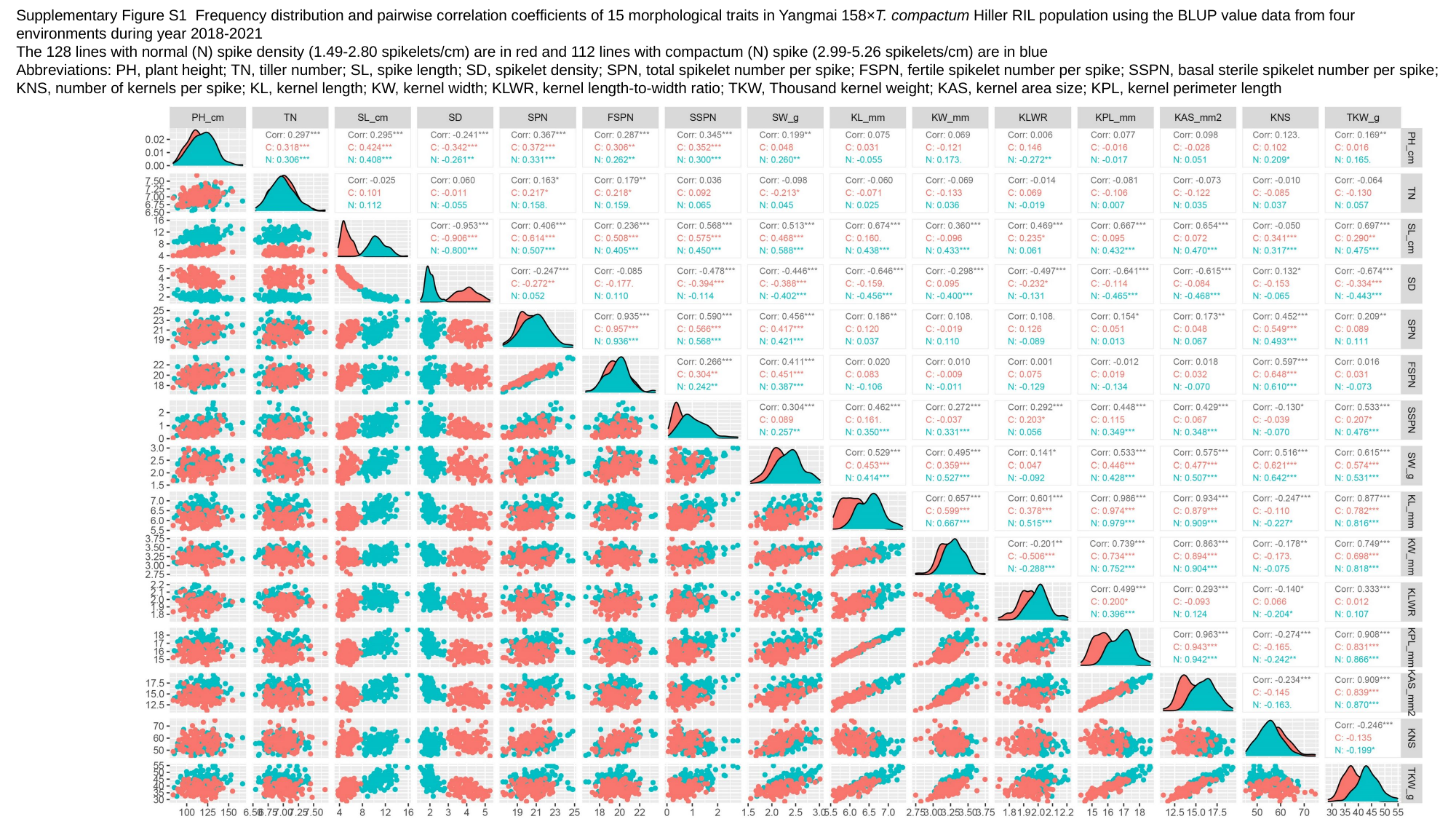

Supplementary Figure S1 Frequency distribution and pairwise correlation coefficients of 15 morphological traits in Yangmai 158×T. compactum Hiller RIL population using the BLUP value data from four environments during year 2018-2021
The 128 lines with normal (N) spike density (1.49-2.80 spikelets/cm) are in red and 112 lines with compactum (N) spike (2.99-5.26 spikelets/cm) are in blue
Abbreviations: PH, plant height; TN, tiller number; SL, spike length; SD, spikelet density; SPN, total spikelet number per spike; FSPN, fertile spikelet number per spike; SSPN, basal sterile spikelet number per spike; KNS, number of kernels per spike; KL, kernel length; KW, kernel width; KLWR, kernel length-to-width ratio; TKW, Thousand kernel weight; KAS, kernel area size; KPL, kernel perimeter length

## Slide 2
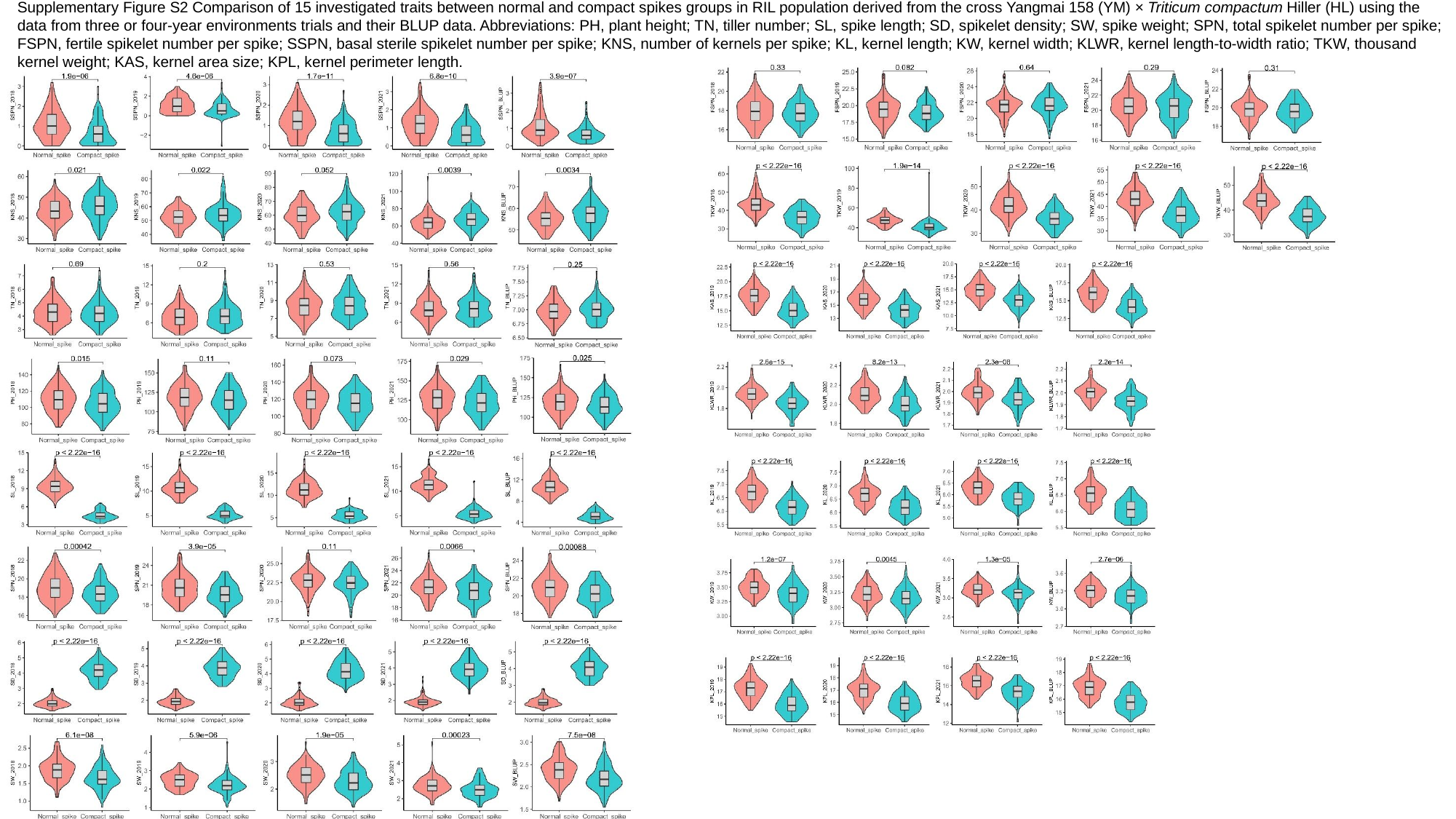

Supplementary Figure S2 Comparison of 15 investigated traits between normal and compact spikes groups in RIL population derived from the cross Yangmai 158 (YM) × Triticum compactum Hiller (HL) using the data from three or four-year environments trials and their BLUP data. Abbreviations: PH, plant height; TN, tiller number; SL, spike length; SD, spikelet density; SW, spike weight; SPN, total spikelet number per spike; FSPN, fertile spikelet number per spike; SSPN, basal sterile spikelet number per spike; KNS, number of kernels per spike; KL, kernel length; KW, kernel width; KLWR, kernel length-to-width ratio; TKW, thousand kernel weight; KAS, kernel area size; KPL, kernel perimeter length.

## Slide 3
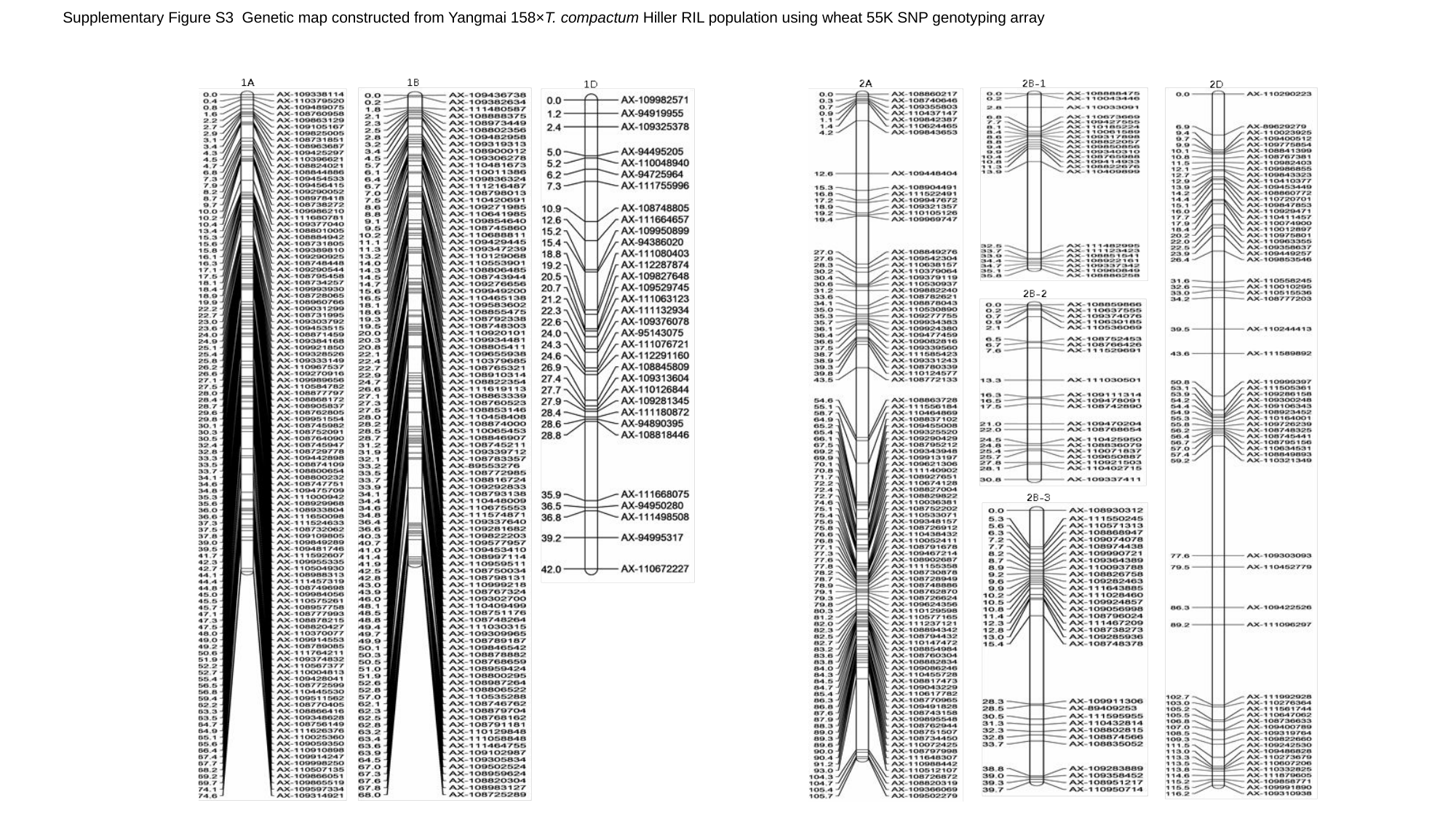

Supplementary Figure S3 Genetic map constructed from Yangmai 158×T. compactum Hiller RIL population using wheat 55K SNP genotyping array

## Slide 4
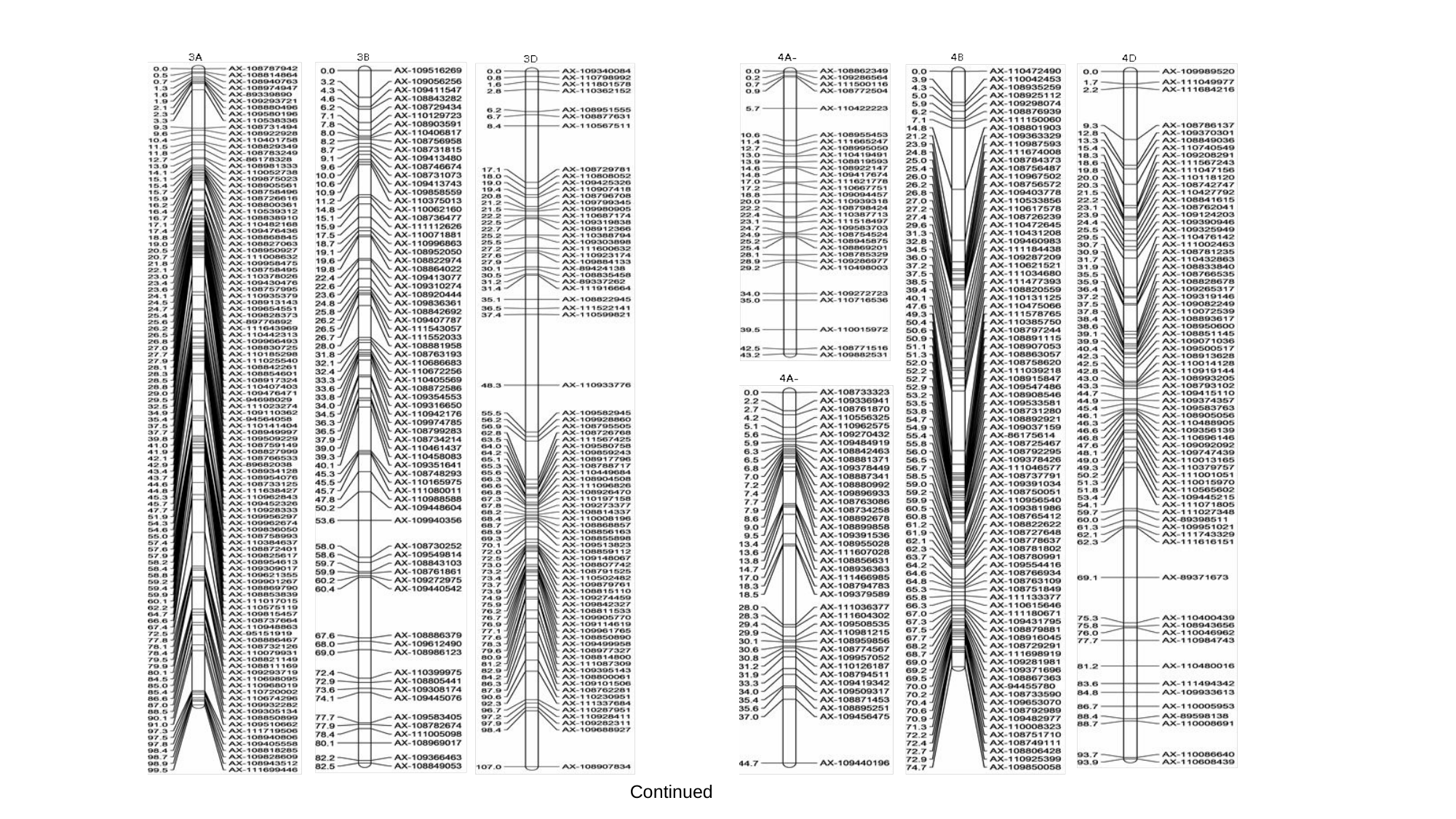

Continued

## Slide 5
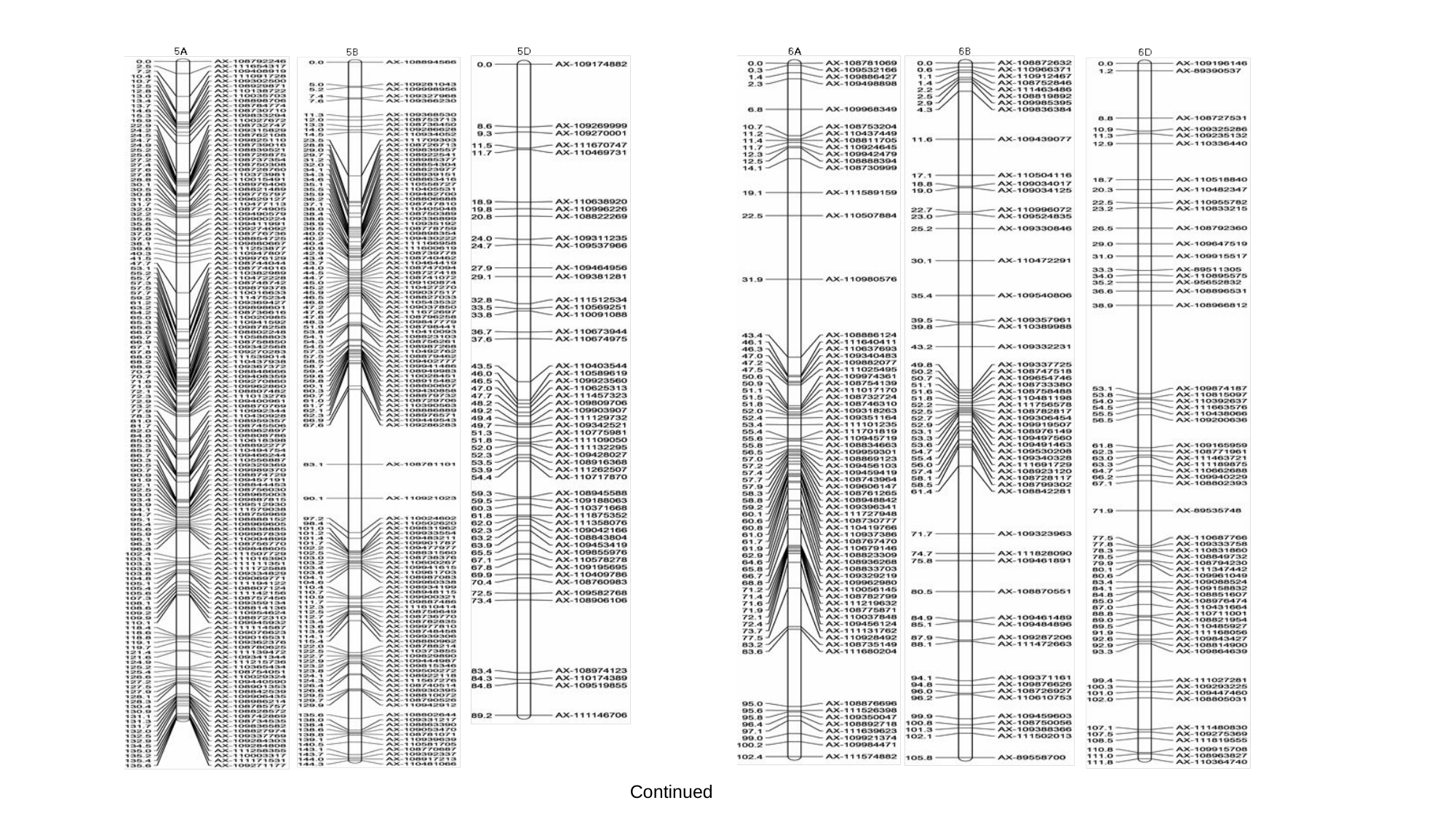

Continued

## Slide 6
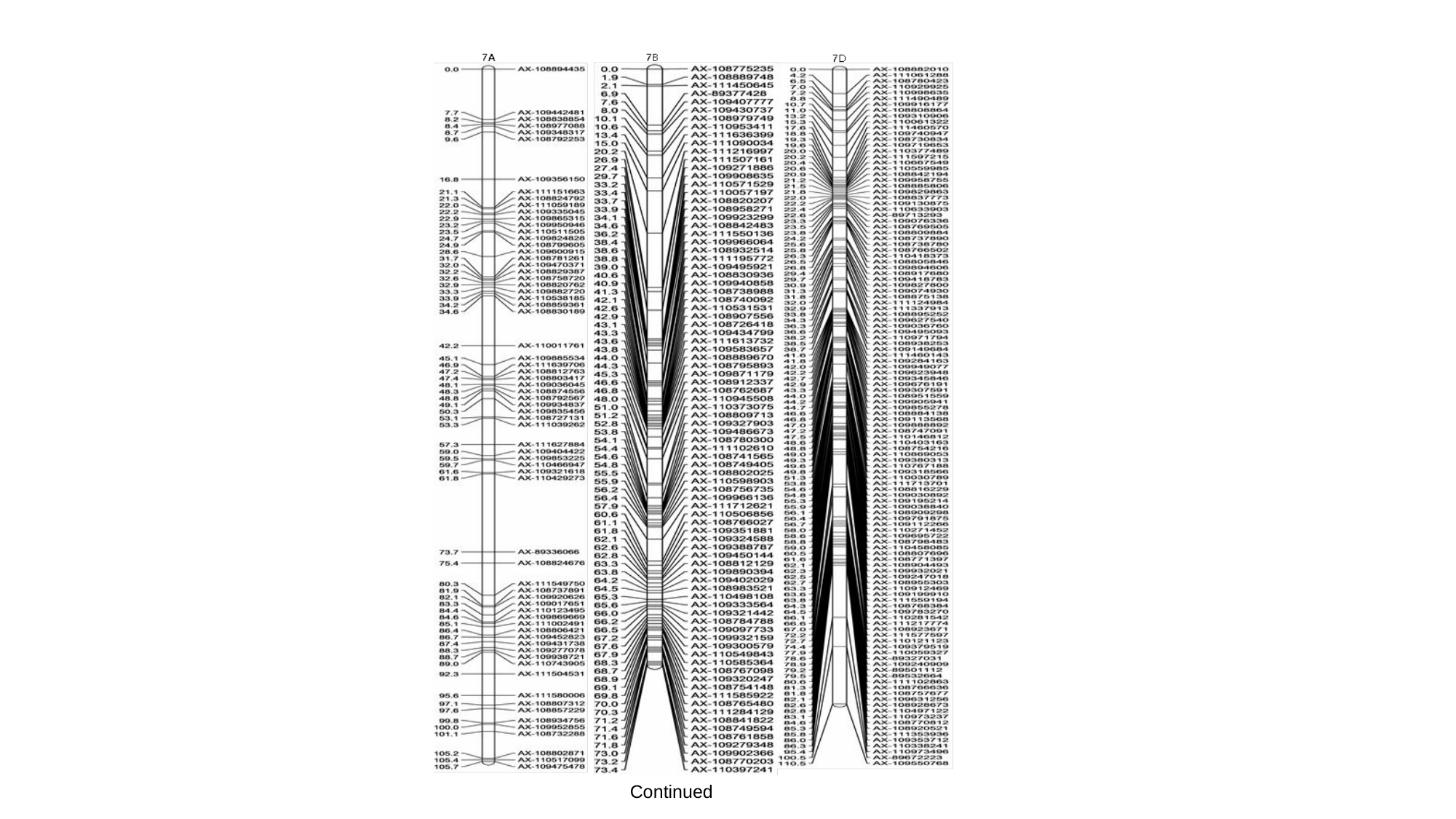

Continued

## Slide 7
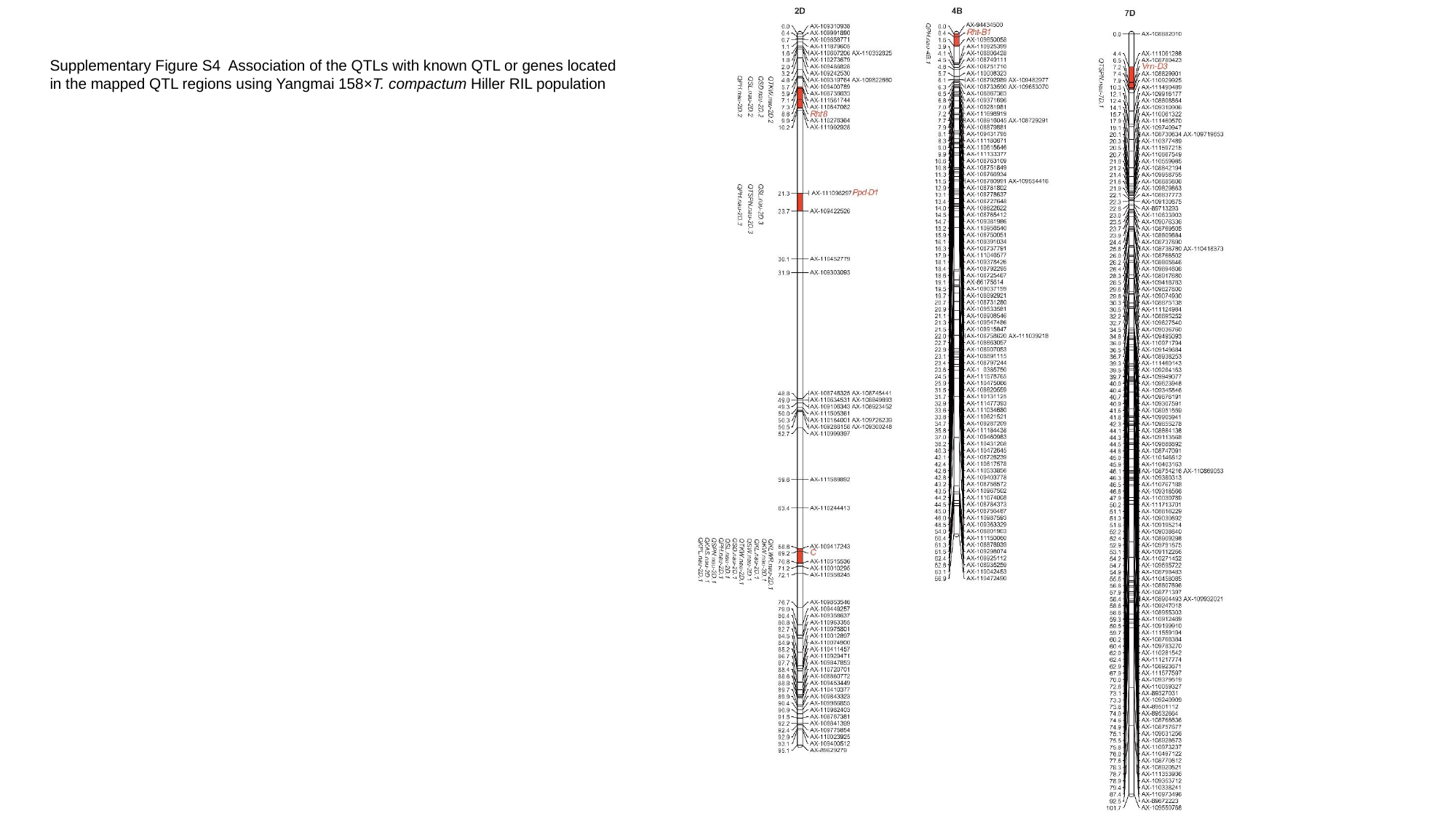

Supplementary Figure S4 Association of the QTLs with known QTL or genes located in the mapped QTL regions using Yangmai 158×T. compactum Hiller RIL population
