# Supplementary material for: Pleiotropic Effect of the compactum Gene and Its Combined Effects with Other Loci for Spike and Grain-Related Traits in Wheat
Source: Plants (Basel). 2022 Jul 13;11(14):1837. doi: 10.3390/plants11141837 (PMC9316965; doi:10.3390/plants11141837)
Supplement: Supplementary file 1 [file plants-11-01837-s001.zip › Supplementary Tables S2&S4.pdf]

Supplementary Table S2 Data for the genetic linkage map constructed using the Yangmai 158/Hiller RIL population

Chromosome	Unique locus	Total SNPs	Length（cM）	cM/locus
1A	109	779	74.6	0.7
1B	101	921	68.0	0.7
1D	33	66	42.0	1.3
2A	98	1782	105.7	1.1
2B-1	22	134	35.8	1.6
2B-2	21	71	30.8	1.5
2B-3	31	141	39.7	1.3
2D	65	275	116.2	1.8
3A	109	757	99.5	0.9
3B	72	606	82.5	1.1
3D	80	322	107.0	1.3
4A-1	31	71	43.2	1.4
4A-2	40	171	44.7	1.1
4B	87	1613	74.7	0.9
4D	73	335	93.9	1.3
5A	144	923	135.6	0.9
5B	121	958	144.3	1.2
5D	52	83	89.2	1.7
6A	70	1150	102.4	1.5
6B	57	648	105.8	1.9
6D	61	186	111.8	1.8
7A	69	254	105.7	1.5
7B	86	909	73.4	0.9
7D	120	748	110.5	0.9
A genome	670	5887	711.4	1.1
B genome	598	6001	655.0	1.1
D genome	484	2015	670.6	1.4
Total	1752	13903	2037.0	1.2

Supplementary Table S4 The primers used in this study

Locus	Marker	Primer name	Sequence (5’→3’)	References
Rht8	*Xgwm261*	gwm261-F	CTCCCTGTACGCCTAAGGC	[35]
		gwm261-R	CTCGCGCTACTAGCCATTG	
Ppd-D1	*M-Ppd-D1*	TaPpd-D1_F1	ACGCCTCCCACTACACTG	[41]
		TaPpd-D1_R1	TGTTGGTTCAAACAGAGAGC	
		TaPpd-D1_R2	CACTGGTGGTAGCTGAGATT	
Rht-B1	-	Rht-B1-F	GAGGTAGGGAGGCGAGAGG	
(cloning)		Rht-B1-R	CGAGAGAGGACGATGAGGAT	
Rht-B1	*M-Rht-B1*	Rht-B-F1	AGGCAAGCAAAAGCTTGAGA	[45]
		Rht-B1a-R2	CCATGGCCATCTCCAGATG	
		Rht-B1b-R2	CCCATGGCCATCTCCAGATA	
Vrn-D3	-	Vrn-D3-F	CTTCTATTCACATGTTTCGTTCATG	
(cloning)		Vrn-D3-R	CCCCCTTACTTAGCCGTTGA	
Vrn-D3	*M-Vrn-D3*	Vrn-D3-F6	CTTCTATTCACATGTTTCGTTCATG	[44]
		Vrn-D3-R8	ACGAGCACGAAGCGATGGATCGC	
